# From peripheral initiation to central integration: a narrative review of the antihypertensive mechanisms of acupuncture in regulating autonomic nervous system homeostasis

**DOI:** 10.3389/fnins.2026.1776680

**Published:** 2026-04-13

**Authors:** Yuting Wang, Zifeng Dai, Linru Hou, Yanyu Yue, Kaixuan Ma, QiuLei Guo, Kc Rachana, Qingguo Liu, Juan Li, Yi Ren, Ruiqi Yao, Jian Wen, Xiaolin Chen, Lili Zhang

**Affiliations:** 1 First Teaching Hospital of Tianjin University of Traditional Chinese Medicine, Tianjin, China; 2 National Clinical Research Center for Chinese Medicine Acupuncture and Moxibustion, Tianjin, China; 3 National Clinical Research Center for Chinese Medicine, Tianjin, China; 4 Beijing University of Chinese Medicine, Beijing, China; 5 School of Health Preservation and Rehabilitation, Chengdu University of Traditional Chinese Medicine, Chengdu, China; 6 Affiliated Sichuan Provincial Rehabilitation Hospital of Chengdu University of Traditional Chinese Medicine, Chengdu, China

**Keywords:** acupuncture, essential hypertension, autonomic nervous system, electroacupuncture, mechanism

## Abstract

Essential Hypertension (EH) is one of the most prevalent chronic cardiovascular diseases, imposing a significant burden on healthcare systems worldwide due to its high rates of disability and mortality. Long-term elevation of blood pressure leads to multi-organ damage in the heart, brain, and kidneys, resulting in severe complications such as coronary heart disease, stroke, and chronic kidney disease. Current treatment for hypertension primarily relies on pharmacological interventions. Although antihypertensive drugs have achieved notable success in controlling blood pressure, challenges remain, including poor long-term medication adherence, side effects, and inadequate blood pressure control in some patients with resistant hypertension. In parallel, acupuncture, a key modality of traditional Chinese medicine, has demonstrated unique advantages in hypertension management in recent years. Characterized by its holistic regulatory effects and minimal side effects, acupuncture is recognized by the World Health Organization as a recommended complementary and alternative therapy for hypertension, although its precise mechanisms remain incompletely understood. This review aims to summarize the “peripheral-central synergy” antihypertensive mechanism of acupuncture in regulating autonomic nervous system (ANS) homeostasis. Studies indicate that acupuncture primarily modulates autonomic homeostasis through the following pathways: (1) activating peripheral nerve fibers to convert physical stimulation into complex bioelectrical signals; (2) regulating synaptic neurotransmitter release and the expression of related membrane receptors; (3) modulating the synaptic microenvironment; (4) regulating the NTS-CVLM-RVLM neural circuit; and (5) modulating the HPA axis neuro-endocrine circuit. Through in-depth analysis, this review elucidates the multi-level and multi-dimensional impact of acupuncture therapy on primary hypertension, providing stronger evidence and a theoretical foundation for its clinical application.

## 1 Introduction

Essential hypertension (EH) represents one of the leading chronic cardiovascular disorders and poses a substantial worldwide public health concern. Data from the 2023 World Hypertension Report highlight a growing global impact, with rising prevalence and death rates each year (Kario et al., 2024). By 2019, around 1.28 billion adults globally lived with hypertension, accounting for nearly 10.8 million deaths per year (Schutte et al., 2023; Zhou et al., 2021a). The resulting high levels of disability and fatality place considerable strain on health systems everywhere. Chronic high blood pressure damages multiple organs, including the heart, brain, and kidneys, often leading to serious conditions like coronary artery disease, stroke, and chronic kidney disease (Zhou et al., 2021b). Notably, autonomic nervous system (ANS) imbalance emerges as both a frequent feature of hypertension and a central driver of its advancement.

Presently, hypertension management depends mainly on medication. Although these drugs successfully lower blood pressure in many cases, persistent issues include low patient compliance over time, adverse reactions, and treatment-resistant forms in certain individuals. Consequently, identifying safe, efficient, and sustainable non-drug complementary approaches has become an important focus in hypertension care.

The ANS holds a pivotal position in managing both immediate blood pressure changes and ongoing stability (Lohmeier and Iliescu, 2015). In healthy states, sympathetic and parasympathetic branches maintain equilibrium to support cardiovascular balance. In hypertensive individuals, however, sympathetic activity often becomes overactive while parasympathetic tone declines, reflected in lower heart rate variability and impaired baroreflex function. This ANS disruption not only stems from hypertension but also serves as a fundamental factor in its onset and continuation.

Acupuncture, a core practice in traditional Chinese medicine, has shown distinct benefits for hypertension treatment in recent studies, offering broad regulatory effects with few unwanted reactions. Recently, several comprehensive reviews have profoundly elucidated the relationship between acupuncture and autonomic nervous system (ANS) modulation. For instance, Fan et al. (2020) summarized the hypotensive role of acupuncture through afferent, central, and efferent pathways, and Li Y. W. et al. (2022) systematically reviewed how acupuncture-induced ANS rebalancing links to its clinical efficacy. While these foundational works establish a macroscopic framework for acupuncture's antihypertensive effects, a detailed, multidimensional mapping that specifically targets synaptic microenvironment modulation (e.g., glia-neurotransmitter crosstalk), precise central neural circuits (e.g., the NTS-CVLM-RVLM loop), and neuroendocrine feedback networks (e.g., the HPA axis) remains to be fully articulated. Against this backdrop, the present review addresses a key question: Does acupuncture primarily influence hypertension by modulating ANS activity? Which precise pathways and molecular processes are involved? Through a detailed synthesis of current evidence, this work seeks to establish a solid theoretical basis for understanding acupuncture's role in hypertension therapy, while providing fresh perspectives and guidance for clinical application and upcoming studies.

## 2 Acupuncture recruits specific Aβ, Aδ, and C afferent fibers to balance autonomic nerve function

Nerve fibers in the autonomic nervous system (ANS) arise from cell bodies in dorsal root ganglia and sympathetic chain ganglia. These fibers are categorized into Aβ, Aδ, and C types according to their conduction speed, myelination degree, and functional roles (Glatte et al., 2019). Group A fibers conduct signals most rapidly, whereas C fibers are the slowest. Each type serves unique purposes and shows distinct anatomical distributions (Siemionow et al., 2011). Sensory A fibers stem from pseudounipolar neurons in dorsal root ganglia and carry inputs to dorsal horn neurons in the spinal cord. After leaving the ganglia, they join motor fibers from the anterior horn, delivering combined sensory and motor supply to peripheral tissues. Both Aβ and Aδ fibers contribute cutaneous sensation. Thickly myelinated Aβ fibers conduct quickly and primarily detect mechanical stimuli, such as touch and vibration (Abraira and Ginty, 2013; Li et al., 2011). Thinner Aδ fibers, with lighter myelination and slower conduction, relay sharp pain, temperature changes, and cold sensations (Abraira and Ginty, 2013; Li et al., 2011; Djouhri, 2016). C fibers include both efferent sympathetic components and sensory afferents. Sympathetic postganglionic C fibers emerge from the lateral horn of spinal segments T8 to L3. These long, unmyelinated fibers pass through gray rami communicantes, integrate into peripheral nerves, and supply blood vessels and piloerector muscles with mainly adrenergic innervation for vasoconstriction, while providing cholinergic signals to sweat glands for perspiration and occasional vasodilation (Al-Horani and Mohammad, 2018; Siepmann et al., 2016). Separate populations of sensory C fibers originate in dorsal root ganglia and innervate skin. These divide into high-threshold mechanoreceptor (C-HTMR) fibers, responsive to painful mechanical, thermal, and chemical stimuli, and low-threshold mechanoreceptor (C-LTMR or C-tactile) fibers, which detect gentle, pleasant touch on hairy and glabrous skin (Roudaut et al., 2012; Campero and Bostock, 2010; Liljencrantz and Olausson, 2014). C-tactile fibers also help modulate pain perception (Habig et al., 2017). Both Aδ and many C fibers respond to multiple stimulus types, earning the label polymodal.

In essential hypertension, abnormal sensory afferent activity plays a critical role in autonomic imbalance, especially heightened sympathetic nerve activity (SNA) (Guyenet et al., 2020). Key examples include: (1) Renal afferents, mostly unmyelinated C fibers, promote widespread SNA elevation in hypertension models, often linked to renal inflammation (Banek et al., 2016; Ong et al., 2019). (2) Reduced arterial baroreceptor sensitivity, where chronic dampening of primarily Aβ-mediated signals contributes to sustained hypertension, as genetic models show that impaired baroreflex input removes normal tonic inhibition of sympathetic outflow (McBryde et al., 2013). (3) Heightened carotid body chemoreceptor afferents (Aδ and C fibers), which display exaggerated responses to hypoxia or chemical stimuli in models like spontaneously hypertensive rats (SHR) and chronic intermittent hypoxia, due to altered ion channel expression (e.g., ASIC3, TASK-1, P2X3) or oxidative stress (Pijacka et al., 2016; Kumar and Prabhakar, 2012; Tan et al., 2010; Nanduri et al., 2019). These overactive afferent inputs converge in the central nervous system, activating sympathetic premotor neurons in the rostral ventrolateral medulla (RVLM) and hypothalamic paraventricular nucleus (PVN), resulting in generalized or organ-targeted sympathetic overdrive that sustains neurogenic hypertension (Nanduri et al., 2019; Stocker et al., 2007; Huber and Schreihofer, 2011; Dampney et al., 2018). Neuropeptides released from C fibers also affect vascular tone. Calcitonin gene-related peptide (CGRP), substance P, and vasoactive intestinal peptide (VIP) trigger vasodilation through their receptors, lowering blood pressure. Capsaicin activates TRPV1 channels on C and Aδ fibers to cause vasodilation, while menthol stimulates TRPM8 channels to induce vasoconstriction, with corresponding effects on blood pressure (Glatte et al., 2019).

The skin's sensory network responds to diverse external and internal stimuli. External triggers include mechanical pressure, chemicals, ultraviolet light, electrical currents, and temperature changes. Internal signals arise from local neuro-immune-endocrine interactions. Structures like hair follicles, arrector pili muscles, and sweat glands receive efferent sympathetic C-fiber innervation. The dermis harbors polymodal Aδ and C fibers acting as nociceptors, thermoreceptors, and mechanoreceptors. These fibers reach the epidermis through free endings and branch to blood vessels, supporting axon reflex responses (Glatte et al., 2019). Acupuncture's mechanical and electrical stimulation thus directly engages these afferents, but the cardiovascular responses are highly dependent on the specific fiber types recruited. Rather than vaguely “activating conduction”, acupuncture precisely recruits specific afferent fibers and evokes action potential discharge within those populations. Foundational electrophysiological studies by Zhou et al. (2005) established critical fiber recruitment thresholds for cardiovascular modulation. Their work demonstrated that while low-intensity stimulation may activate thickly myelinated Aβ fibers, the activation of Aβ afferents alone lacks sufficient empirical support to drive robust cardiovascular responses. Instead, meaningful autonomic modulation occurs only when stimulation intensity surpasses the activation thresholds for thinly myelinated Aδ (Group III) and unmyelinated C (Group IV) fibers, with greater intensity yielding stronger effects (Zhou et al., 2005; Li et al., 2007).

**Figure 1:**
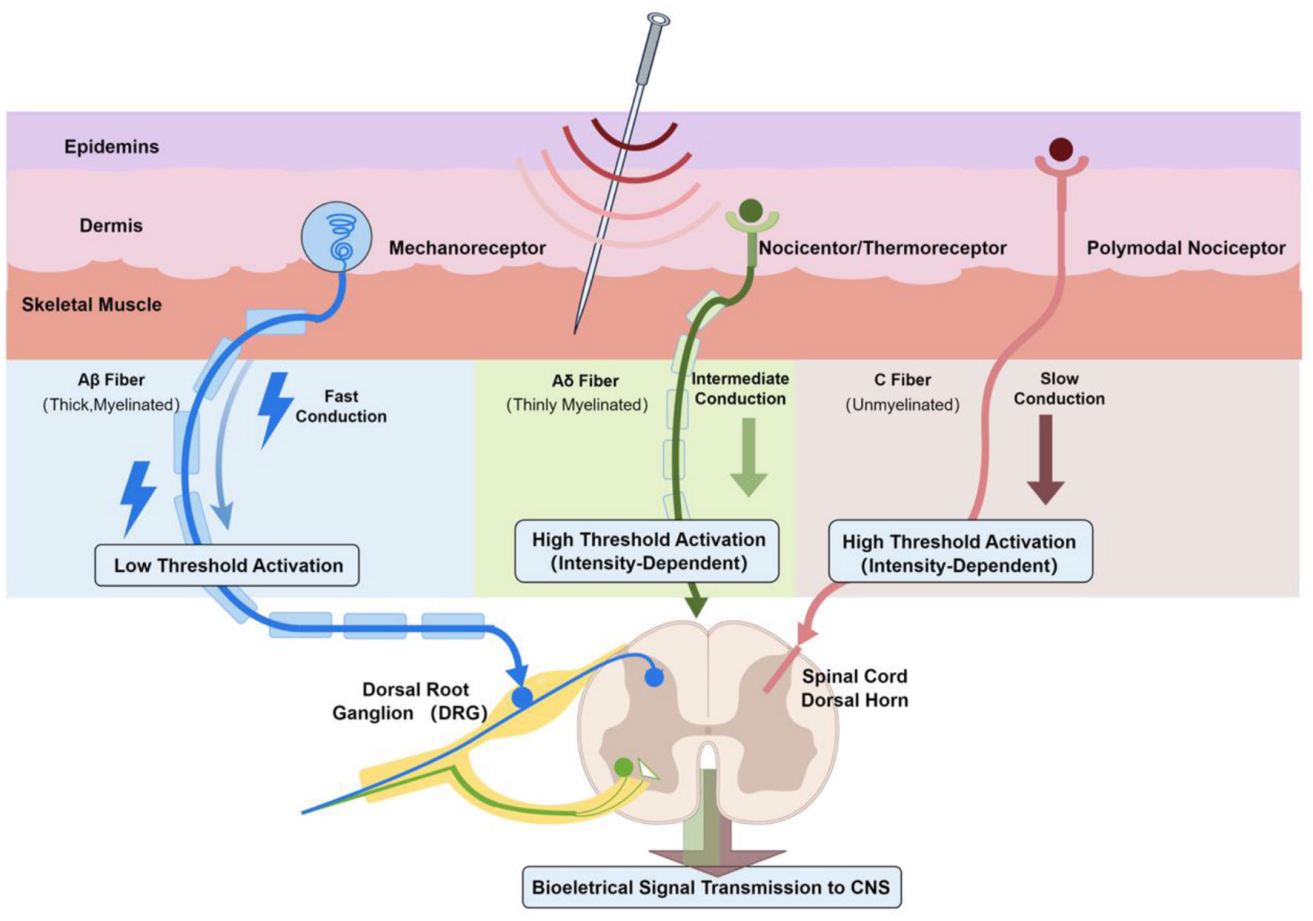
Peripheral mechanisms of acupuncture: selective activation of sensory nerve fibers. *Note* While Aβ fibers mediate low-threshold mechanosensation, the robust modulation of central autonomic cardiovascular networks primarily depends on the intensity-dependent recruitment of Aδ and C fibers.

In SHR experiments, electrical stimulation of the sciatic nerve or gastrocnemius muscle produced prolonged SNA suppression and blood pressure reduction reversible by naloxone, highlighting opioid-mediated sympathetic inhibition (Thorén et al., 1990). Similar work shows acupuncture at median nerve sites activates Group III (Aδ-equivalent) and Group IV (C-equivalent) afferents, dampening pressor reflexes via central autonomic circuits (Tjen-A-Looi et al., 2005). Detection of phosphorylated ERK in dorsal root ganglia further confirms that acupuncture powerfully excites these primary sensory neurons (Chen et al., 2008), clarifying how it recruits peripheral Aδ and C afferents to influence autonomic regulation (Guo et al., 2018). Therefore, while Aβ fibers may contribute to initial mechanosensation (e.g., the “Deqi” sensation), the recruitment of Aδ and C fibers is the essential neurophysiological prerequisite for acupuncture's antihypertensive effects (Figure 1).

## 3 Acupuncture regulates autonomic nerve function by modulating synapses of central neurons

### 3.1 Acupuncture modulates neurotransmitter transmission by regulating receptor expression on central neuronal synaptic membranes

The baroreflex pathway depends on intact synaptic function to maintain proper blood pressure control. When arterial pressure increases, mechanoreceptors in the carotid sinus and aortic arch detect vessel wall stretch and generate afferent signals. These impulses travel through cranial nerves to the brainstem, primarily reaching the nucleus of the solitary tract (NTS), which serves as the initial integration site (Wan et al., 2025; Hikosaka et al., 2022). In healthy conditions, NTS activation stimulates inhibitory neurons in the caudal ventrolateral medulla (CVLM). These CVLM neurons, in turn, suppress activity in the rostral ventrolateral medulla (RVLM), the main sympathoexcitatory center, thereby decreasing sympathetic outflow to the cardiovascular system. Simultaneously, NTS projections enhance parasympathetic tone by exciting neurons in the dorsal motor nucleus of the vagus (DMV) and nucleus ambiguus, promoting vagal bradycardia and vasodilation. At the synaptic level, the postsynaptic membrane acts as the key integration hub, determining neuronal excitability and output based on incoming signals. Presynaptic terminals release neurotransmitters into the synaptic cleft, while postsynaptic elements detect and transduce these molecules via receptors, ion channels, and intracellular cascades, ultimately deciding whether to generate an action potential. Synaptic communication extends beyond simple unidirectional transfer; it forms a dynamic, multidimensional network with extensive feedback and crosstalk. Neurotransmitters such as gamma-aminobutyric acid (GABA), endogenous opioids, and glutamate bind to autoreceptors and heteroreceptors on both presynaptic and postsynaptic sides, creating sophisticated regulatory loops. Activation of presynaptic autoreceptors typically triggers negative feedback, limiting further release of the same transmitter. Binding to presynaptic heteroreceptors from adjacent terminals of different neuronal populations can similarly suppress release of other neurotransmitters, enabling precise cross-system modulation.

#### 3.1.1 Acupuncture regulates the expression of GABAA/GABAB receptors

GABA serves as the primary inhibitory neurotransmitter in the central nervous system. The GABAA receptor, a ligand-gated chloride channel, appears extensively on postsynaptic membranes in key cardiovascular regions, including the rostral ventrolateral medulla (RVLM) and nucleus of the solitary tract (NTS) (Saha et al., 2001; Coleman and Dampney, 1998). Upon GABA binding, the channel opens, allowing chloride ions to enter the cell. This causes hyperpolarization of the postsynaptic membrane, producing an inhibitory postsynaptic potential (IPSP) that lowers neuronal firing rates (Dembitskaya et al., 2025). The GABAB receptor, a G-protein-coupled metabotropic type, mediates slower inhibition. It exists on postsynaptic sites for direct IPSPs and as presynaptic auto- or heteroreceptors. As a heteroreceptor on terminals of other neurons, often glutamatergic, it modulates transmitter release. GABA activation of these presynaptic GABAB sites engages G-proteins, releasing Gβγ subunits that block voltage-gated calcium channels, reducing calcium entry, and then curbs excitatory neurotransmitter release, such as glutamate.

First, the NTS acts as the main integration hub for peripheral cardiovascular signals, vital for overall autonomic stability (Lima-Silveira et al., 2022; Machado et al., 1997; Lawrence and Jarrott, 1996). Evidence shows heightened GABAergic tone in the NTS promotes hypertension development (Potts, 2006; Zhang and Mifflin, 2010; Zubcevic and Potts, 2010). Excessive inhibition here impairs the baroreflex, a core pressure-stabilizing pathway. Overactive GABA signaling blocks depressor signals at the NTS, leading to RVLM disinhibition, increased sympathetic drive, and persistent blood pressure elevation. Acupuncture plays a key role in adjusting GABA receptor expression and activity in the brainstem NTS, likely contributing to enhanced baroreflex function and pressure reduction (Lima-Silveira et al., 2022). In renovascular hypertensive rats (2K1C model), NTS GABAB receptors showed compensatory upregulation with exaggerated responses to local drugs, indicating disrupted transmission. Daily electroacupuncture at ST36 (Zusanli) for 2 weeks reversed these changes: it lowered arterial pressure, improved baroreflex sensitivity, normalized drug responses, and reduced elevated GABAB expression (Zhang et al., 2018). This highlights acupuncture's homeostatic approach normalizing pathologically high receptor levels to restore balanced NTS inhibition and alleviate sympathetic overactivity.

Second, acupuncture fine-tunes medullary cardiovascular control via GABAA receptors across interconnected nuclei. The RVLM functions as the primary sympathetic output center, while the caudal ventrolateral medulla (CVLM) contains essential inhibitory interneurons (Kumagai et al., 2012; Guyenet et al., 2018). During sympathoexcitatory states, electroacupuncture effectively engages these medullary circuits to counteract sympathetic overactivity. Tracing studies have identified direct NTS-to-RVLM pathways activated by specific acupoints, such as PC6 (Neiguan), likely involving GABAergic transmission to inhibit the sympathetic final path (Guo and Malik, 2019). At higher centers, such as the hypothalamic arcuate nucleus (ARC), acupuncture triggers prolonged descending inhibition targeting RVLM GABAA receptors for lasting antihypertensive effects. In cat models, electroacupuncture stimulation at PC6 (Neiguan) suppression of bradykinin-evoked pressor responses lasted over an hour, depending on ARC excitatory input. Late blockade of RVLM GABAA receptors promptly reversed this sustained inhibition, confirming ongoing GABAergic tone on RVLM neurons as the downstream effector (Tjen-A-Looi et al., 2007). This bridges short- and long-term acupuncture actions by enhancing brainstem inhibition through higher-center-driven mechanisms. In the dorsal motor nucleus of the vagus (DMV), electroacupuncture increases parasympathetic output by reducing GABAA receptor expression, thereby relieving inhibition of vagal neurons (Yang et al., 2021). Such bidirectional GABA modulation, strengthening inhibition where needed (e.g., RVLM) and weakening it elsewhere (e.g., DMV), underlines acupuncture's refined strategy for rebalancing autonomic function.

#### 3.1.2 Acupuncture regulates the expression of μ- and δ-opioid receptors

Endogenous opioid peptides, including enkephalins, endorphins, and dynorphins, rank among the key neuromodulators in the central nervous system. Opioid receptors μ (mu), δ (delta), and κ (kappa) belong to the G-protein-coupled receptor family and appear extensively on presynaptic terminals and postsynaptic membranes in cardiovascular regulatory areas, such as the rostral ventrolateral medulla (RVLM) and nucleus of the solitary tract (NTS). Binding of these peptides to postsynaptic μ or δ receptors activates Gi/o signaling, increasing potassium efflux. This hyperpolarizes the postsynaptic membrane, creates a prolonged inhibitory postsynaptic potential, and decreases neuronal excitability and discharge rates. A primary role emerges at presynaptic sites, where opioid receptors function as heteroreceptors on terminals releasing GABA, glutamate, or other transmitters. Activation triggers Gi/o pathways that block voltage-gated calcium channels and open potassium channels, limiting calcium entry during action potentials and reducing neurotransmitter release. This presynaptic inhibition enables precise control over synaptic strength in interconnected systems. In the RVLM, μ- and δ-opioid receptors drive central inhibitory actions of acupuncture. Cat studies showed that electroacupuncture at Neiguan (PC6) and Jianshi (PC5) blockade of pressor reflexes from gallbladder stimulation reversed upon local naloxone injection into the RVLM, establishing these receptors as essential mediators (Li et al., 2001). Electrophysiological data further indicated prolonged dampening of RVLM presympathetic neuron responses to visceral inputs, quickly undone by naloxone (Tjen-A-Looi et al., 2003). Colocalization studies revealed δ-receptors on glutamatergic cells and synapses, where electroacupuncture at Neiguan (PC6) and Jianshi (PC5) curbs bradykinin-triggered sympathetic reflexes by opioid suppression of glutamate signaling (Zhou et al., 2007). Acupuncture influence on the opioid system stands out for its prolonged duration and acupoint precision, matching observed clinical persistence and site-specific effects. Repeated electroacupuncture at Neiguan (PC6) and Jianshi (PC5) boosts enkephalin gene and protein levels in the RVLM for days, explaining sustained benefits post-treatment (Li et al., 2012). In cold-stress hypertension models, intra-RVLM injection of a δ-antagonist partially reversed the pressure-lowering effects of electroacupuncture at Zusanli (ST36) and Shangjuxu (ST37), while δ-agonist administration replicated the depressor response (Li et al., 2016). This confirms elevated enkephalin acts via postsynaptic δ-receptors to exert antihypertensive actions.

Furthermore, acupuncture displays profound acupoint and stimulation-depth selectivity in modulating autonomic reflexes. For instance, deep nerve stimulation (engaging muscle afferents) at ST36 (Zusanli) and PC6 (Neiguan) robustly recruits μ-opioid mechanisms in the NTS to buffer cardiovascular reflexes, whereas superficial cutaneous stimulation produces no such central effect (Tjen-A-Looi et al., 2018). Although this specific study demonstrated the buffering of reflex hypotension, it reveals a crucial neuroanatomical principle for hypertension management: the activation of NTS opioid networks requires precise, deep somatic inputs. Once engaged, this same NTS endogenous opioid system acts as a vital central brake to dampen exaggerated sympathetic outflows, thereby providing the neurochemical foundation for acupuncture's antihypertensive effects.

**Figure 2:**
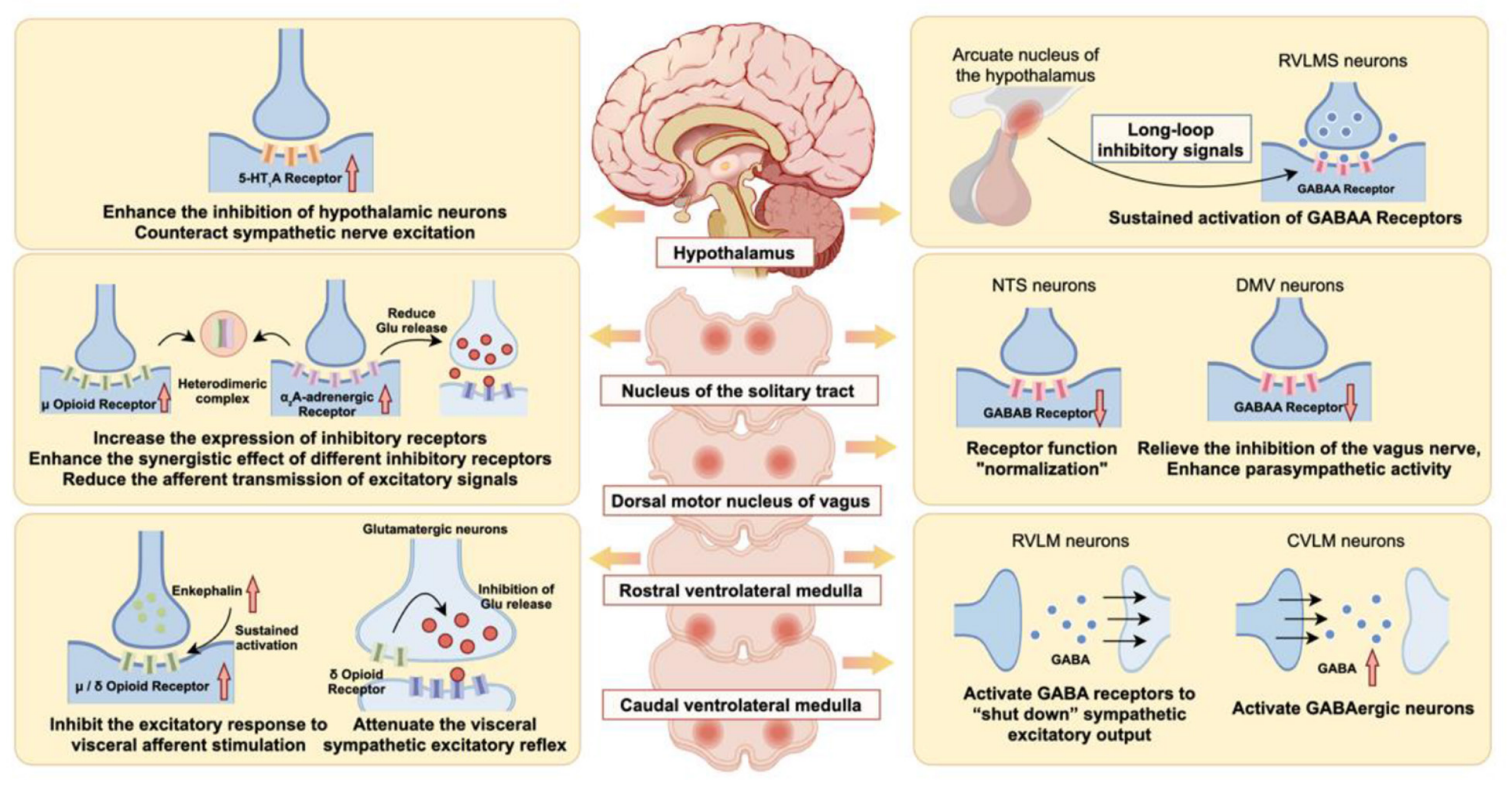
“Inhibitory receptor synergy-GABA brake” mechanism of acupuncture: activation of the composite regulation of 5-HT1A, μ/δ-opioid receptors and α_2a_-adrenoceptors, enhancement of GABAA-mediated long-loop inhibition, suppression of glutamatergic excitatory input, downregulation of sympathetic output from the rostral ventrolateral medulla (RVLM), and elevation of vagal/parasympathetic tone.

Acupuncture also displays acupoint selectivity: deep nerve stimulation at ST36 (Zusanli) and PC6 (Neiguan) inhibits reflex hypotension and bradycardia through μ-opioid mechanisms in the NTS, but stimulation of points engaging only superficial cutaneous afferents yields no effect (Tjen-A-Looi et al., 2018). Such specificity underscores how acupuncture engages particular somatic afferents to activate central endogenous opioid pathways for pain relief and blood pressure control (Figure 2).

#### 3.1.3 Acupuncture regulates the expression of N-Methyl-D-aspartate (NMDA) receptors

**Figure 3:**
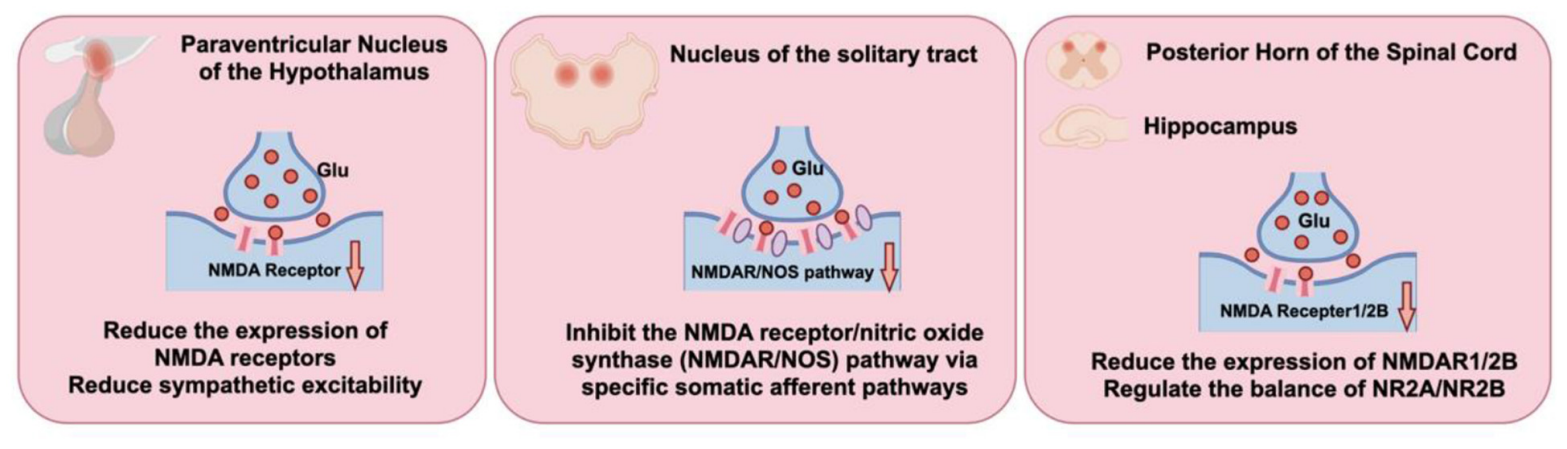
NMDA-glutamate excitability downregulation mechanism of acupuncture: reduction of NMDA receptor expression/activity in key nuclei such as the paraventricular nucleus of the hypothalamus (PVN) and nucleus tractus solitarius (NTS), inhibition of the NMDAR-nitric oxide synthase (NOS) pathway, as well as downregulation of NMDAR1/2B and modulation of the NR2A/NR2B balance in the dorsal horn of the spinal cord and hippocampus, thereby globally attenuating sympathetic excitability and central sensitization.

Glutamate acts as the main excitatory neurotransmitter in the central nervous system. Ionotropic AMPA and NMDA receptors, both ligand-gated cation channels, appear extensively on postsynaptic membranes in cardiovascular control areas, including the rostral ventrolateral medulla (RVLM) and nucleus of the solitary tract (NTS). Glutamate binding to AMPA receptors opens channels for rapid sodium influx, depolarizing the postsynaptic membrane and producing a quick excitatory postsynaptic potential (EPSP) that boosts neuronal firing. This initial depolarization removes the magnesium block from NMDA channels. Subsequent glutamate activation allows significant calcium entry, creating a prolonged EPSP and triggering intricate intracellular signaling cascades. Metabotropic glutamate receptors (mGluRs), G-protein-coupled, occur on postsynaptic sites for slower modulation and as presynaptic auto- or heteroreceptors. As heteroreceptors, often on GABAergic terminals Group II mGluR activation by spillover glutamate engages Gi/o proteins, releasing Gβγ subunits that block voltage-gated calcium channels. Reduced calcium influx then limits GABA release. As autoreceptors on glutamatergic terminals, these receptors provide essential negative feedback: excess glutamate curbs further release through the same pathway, preventing overexcitation and potential toxicity while lowering overall glutamatergic transmission. NMDA receptors stand out as critical excitatory players in the CNS, with overactivity linked to elevated sympathetic nerve activity (SNA) in cardiovascular disorders (Soda et al., 2023). In hypertension development, excessive NMDA signaling in autonomic hubs like the hypothalamus and medulla drives ongoing sympathetic activation. Studies show acupuncture at Zusanli (ST36) effectively adjusts NMDA receptor expression and activity across levels, dampening central excitation and reestablishing autonomic equilibrium (Tahir et al., 2024; Ma et al., 2022). Basic investigations reveal heightened NMDA function as a major contributor to sympathetic overdrive and chronic hypertension. In the hypothalamic paraventricular nucleus (PVN), prolonged stress elevates the accessory protein α2δ-1, enhancing NMDA receptor synaptic delivery and function to maintain sympathetic outflow and high pressure (Zhou J. J. et al., 2021). This identifies a prime target for acupuncture. Electroacupuncture reliably reduces overexpressed NMDA receptors in vital cardiovascular regions. In spontaneously hypertensive rats (SHR), treatment markedly lowered PVN NMDA receptor levels, alongside reduced pressure and sympathetic tone. Combining electroacupuncture at Taichong (LR3) and Quchi (LI11) with an NMDA antagonist yielded stronger antihypertensive results than acupuncture alone (Jiao et al., 2025). These observations indicate NMDA pathway suppression as a central mechanism for electroacupuncture benefits, potentially amplifying natural inhibitory processes. The modulation shows clear acupoint dependence: in nicotine-triggered hypothalamic norepinephrine models, stimulation at HT7 (Shenmen) suppressed the NMDA/nitric oxide synthase cascade in the NTS, but PC6 or non-acupoint sites did not (Liu et al., 2019). This demonstrates how acupuncture uses selective somatic inputs to target NMDA activity in specific brain nuclei precisely (Figure 3).

Furthermore, acupuncture modulation of NMDA receptors is not confined to cardiovascular centers but exhibits a similar inhibitory pattern in the spinal cord and higher brain regions, reflecting its broad central regulatory capacity. In a spinal cord injury model, electroacupuncture treatment at Dazhui (GV14) and Mingmen (GV4) significantly reduced the abnormally elevated expression of NR1 and NR2 subunits of NMDA receptors (Tu et al., 2017). In visceral hypersensitivity associated with irritable bowel syndrome, the alleviating effect of electroacupuncture at Zusanli (ST36) and Shangjuxu (ST37) was closely linked to the downregulation of NR2B subunit expression (Liu et al., 2017). Similarly, in studies on vascular dementia and the hippocampus, electroacupuncture intervention at Baihui (GV20) and Shenting (GV24) (Jiang et al., 2020) or at Baihui (GV20), Shenshu (BL23) and Zusanli (ST36) (Meng et al., 2008), also robustly reduced NMDAR1 expression. Collectively, these consistent findings across different central regions and pathological models demonstrate that downregulating abnormally heightened NMDA receptor expression is a universal and key molecular event underlying acupuncture's central inhibitory effects.

#### 3.1.4 Acupuncture regulates the expression of 5-HT1A receptors

Serotonin (5-hydroxytryptamine, 5-HT) ranks as a vital neuromodulator in the central nervous system. The 5-HT_3_ receptor, a ligand-gated cation channel, occurs extensively on postsynaptic membranes in cardiovascular regulatory sites, including the nucleus of the solitary tract (NTS) and area postrema. Serotonin binding opens the channel, mainly allowing sodium influx. This depolarizes the postsynaptic membrane, creates a rapid excitatory postsynaptic potential (EPSP), and elevates neuronal discharge rates. Other subtypes, such as 5-HT_1_ and 5-HT_2_, are G-protein-coupled metabotropic receptors. These are widely distributed at postsynaptic sites, mediating slower potentials: 5-HT_1_ subtypes generally inhibit, while 5-HT_2_ subtypes excite. They also serve as presynaptic auto- and heteroreceptors. As heteroreceptors often on GABAergic or glutamatergic terminals activation by serotonin (e.g., via 5-HT_1_A or 5-HT_2_A) triggers Gi/o or Gq pathways. This alters voltage-gated calcium channels or potassium channels, curbing calcium entry or promoting hyperpolarization, which suppresses or enhances release of GABA or glutamate accordingly. As autoreceptors on serotonergic terminals (e.g., 5-HT_1_B/_1_D), they deliver negative feedback: excess serotonin activates these sites, engaging G-protein cascades to limit further serotonin release. This acts as an essential control mechanism to maintain balanced serotonergic signaling. In the serotonergic pathway, acupuncture exhibits refined bidirectional modulation, boosting inhibitory receptors while suppressing excitatory ones. Gene array analyses in spontaneously hypertensive rats (SHRs) demonstrated a downregulated hypothalamic expression of the inhibitory 5-HT1A receptor; however, electroacupuncture at the ST9 (Renying) acupoint markedly upregulated this expression (Guo et al., 2017). This classic inhibitory receptor, when engaged, opens potassium channels for hyperpolarization, countering sympathetic overactivity (Villas-Boas et al., 2021). By dampening this central sympathetic drive, the upregulation of 5-HT1A receptors directly contributes to a reduction in peripheral vascular resistance and cardiac workload, thereby translating into a clear antihypertensive effect (Guyenet et al., 2018, 2020). At the same time, acupuncture dampens activity of excitatory serotonin receptor subtypes, further synergizing to lower elevated blood pressure (Figure 2).

#### 3.1.5 Acupuncture regulates the expression of α2A-AR

In the adrenergic pathway, acupuncture primarily strengthens central inhibitory processes, with a focus on the α_2_A-adrenergic receptor (α_2_A-AR). This receptor operates mainly as a presynaptic autoreceptor and postsynaptic inhibitor. Studies show that electroacupuncture at LR3 (Taichong) on the Liver meridian effectively lowers blood pressure and sympathetic nerve activity in spontaneously hypertensive rats while specifically increasing α_2_A-AR levels in the nucleus of the solitary tract (NTS) (Zhang et al., 2021). The NTS serves as the key integration hub for cardiovascular reflexes, and higher α_2_A-AR expression there boosts inhibitory tone. This occurs through presynaptic reduction of excitatory transmitters like glutamate and direct postsynaptic suppression of neuronal activity (Shields et al., 2009; Ohshima et al., 2017). A potential molecular basis involves interactions with μ-opioid receptor/α_2_A-AR heterodimers (Zhang et al., 2021), highlighting crosstalk among inhibitory networks (Figure 2).

#### 3.1.6 Acupuncture regulates the expression of NK1 receptors

Substance P (SP) and its primary receptor, the neurokinin 1 (NK1) receptor, play key roles in mediating stress responses, pain transmission, and inflammation factors that often promote sympathetic activation (Steinhoff et al., 2014; Humes et al., 2024). The NK1 receptor, a G-protein-coupled type, activates the Gq pathway upon ligand binding, stimulating phospholipase C to produce inositol trisphosphate (IP_3_) and diacylglycerol (DAG). This triggers intracellular calcium mobilization and neuronal excitation (Douglas and Leeman, 2011; Dittrich et al., 2012). Evidence from exercise studies shows that muscle afferents release SP during physical activity, exciting GABAergic neurons in the nucleus of the solitary tract (NTS); blocking NK1 receptors there with antagonists reduces post-exercise blood pressure drops (Chen et al., 2009). This process offers a useful parallel for acupuncture, which also delivers somatic sensory stimulation. A reasonable hypothesis emerges: acupuncture might trigger similar pathways, promoting internalization or reduced expression of NK1 receptors in critical areas like the NTS and hypothalamic paraventricular nucleus (PVN). Such changes could diminish SP-driven excitatory and stress-related signaling, helping curb sympathetic nerve activity and lower blood pressure. Overall, while direct investigations into acupuncture's effects on NK1 receptors remain limited, supporting indirect findings point to this receptor as a promising new player in central cardiovascular control. Upcoming research exploring whether acupuncture restores autonomic equilibrium by altering NK1 receptor expression or activity could uncover fresh insights into its brainstem and hypothalamic mechanisms.

### 3.2 Acupuncture modulates the synaptic cleft “environment” to influence synaptic transmission

The synaptic cleft serves as a vital junction for neuronal communication, where its dimensions and structural features directly influence transmission efficiency. This narrow space contains elements like microfilaments, intersynaptic threads, and S-100 proteins. Abnormal widening of the cleft can disrupt component synthesis, impair neurotransmitter binding, hinder S-100 interactions with calcium ions, and ultimately impede impulse propagation. In healthy conditions, neurotransmitters undergo swift removal after signaling to ensure tight control over synaptic strength. Acetylcholine breaks down via cholinesterase; monoamines including norepinephrine, dopamine, and serotonin face partial degradation inside target cells by enzymes such as catechol-O-methyltransferase and monoamine oxidase, with the remainder reabsorbed through presynaptic uptake for recycling. Peptide transmitters mainly inactivate through proteolysis by aminopeptidases, carboxypeptidases, and endopeptidases. A minor portion diffuses into surrounding fluid, becoming diluted and inactive. Glial cells increasingly gain recognition for their involvement in axon pruning and synapse elimination, where they actively engulf synaptic components and debris. Key types astrocytes, microglia, and oligodendrocyte precursors all play essential roles in refining synapses across the mammalian central nervous system.

#### 3.2.1 Acupuncture modulates glutamate concentration in the synaptic cleft

**Figure 4:**
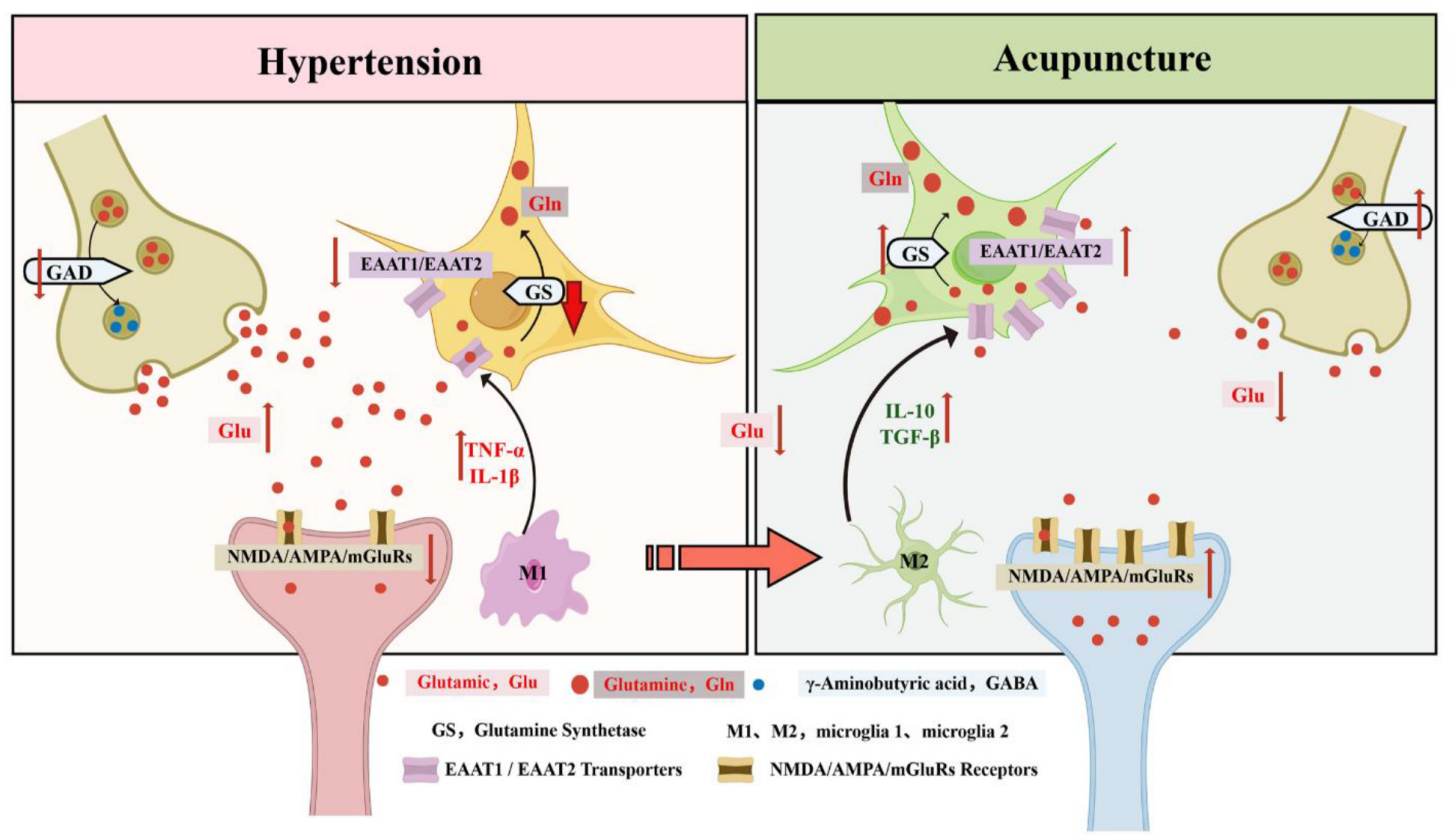
Glutamate-glutamine cycle mechanism of acupuncture: selective activation of astrocyte EAAT1/EAAT2 and GS-mediated glutamate clearance and recycling, inhibition of postsynaptic NMDA/AMPA/mGluRs overexcitation and M1-type inflammatory response, and attenuation of excessive excitatory synaptic transmission associated with hypertension.

Glutamate serves as the chief excitatory neurotransmitter in the central nervous system, participating in over half of all synaptic events (Parkin et al., 2018). Efficient removal from the synaptic space proves essential for normal neural activity and protection against damage (Vazquez-Juarez et al., 2023; Huang et al., 2018). Delayed clearance allows buildup, overstimulating receptors and sparking excitotoxic processes: disrupted calcium balance, elevated nitric oxide, activated proteases, and eventual cell injury or loss (Verma et al., 2022; Yang et al., 2019). Astrocytes form extensive signaling networks with neurons, facilitating brain communication. The glutamatergic tripartite synapse encompassing presynaptic terminal, postsynaptic neuron, and surrounding astrocyte stands central to glutamate handling in mammals (Machado-Vieira et al., 2009). Presynaptic neurons release glutamate, which acts on ionotropic and metabotropic receptors postsynaptically. Excess levels trigger feedback, boosting astrocytic uptake via EAAT1 and EAAT2 transporters and enhancing clearance to stabilize synaptic function (Banasr and Duman, 2008). Inside astrocytes, glutamine synthetase (GS) converts glutamate to glutamine, a pivotal step in the glutamate-glutamine shuttle that reflects metabolic efficiency (Ozawa, 2007). Astrocytic EAAT1 and EAAT2 handle 80–90% of released glutamate, keeping extracellular concentrations low to avoid toxicity; dysfunction links to neurologic conditions (Fontana et al., 2023; Wilkie et al., 2021). In intracerebral hemorrhage models using autologous blood injection, primary motor cortex showed reduced EAAT2 expression and activity, causing glutamate buildup, excitotoxicity, neuronal loss, and movement deficits—potentially disrupting autonomic pathways. Electroacupuncture at LI11 (Quchi) and ST36 (Zusanli) restored EAAT2 protein and mRNA levels, improved clearance, lessened damage, enhanced motor recovery, and likely stabilized autonomic regulation through repaired circuits (Chen et al., 2025). Chronic unpredictable mild stress models revealed prefrontal cortex benefits from electroacupuncture at LR3 (Taichong) and LI4 (Hegu), increasing astrocytic EAAT1, EAAT2, and GS to bolster glutamate uptake (Luo et al., 2016). In middle cerebral artery occlusion (MCAO) stroke models, animals developed spasticity, glutamate excess, GABA decline, and autonomic irregularities (e.g., altered heart rate variability). Acupuncture activated GLT-1/EAAT2, rebalancing the glutamate/GABA-glutamine cycle, normalizing excitatory-inhibitory transmission, and easing hyperexcitability-induced spasms (Zhang Z. et al., 2025). Microglia act as the CNS's main phagocytes, removing debris and dying cells under normal conditions (Wang et al., 2021). They adapt to signals by shifting polarization states for immune roles, though pathology can turn protective functions harmful (Kaur et al., 2017). In resting states, microglia display branched (ramified) shapes, surveying the environment and pruning synapses. Activation transforms them to rounded (amoeboid) forms with heightened phagocytosis and inflammatory involvement. Polarization yields M1 (pro-inflammatory, releasing TNF-α, IL-1β, IL-6, and oxidants to worsen inflammation) or M2 (anti-inflammatory, secreting protective factors to aid repair) phenotypes (Zhang et al., 2024b). Stimuli like ATP trigger glutamate release from microglia via P2X7-driven exchange, caspase-mediated exocytosis, or pannexin-1 leakage (Sidoryk-Wegrzynowicz and Strużyńska, 2021). Microglial cytokines (e.g., IL-1β, TNF-α) suppress astrocytic GLT-1/GLAST, impairing uptake and raising extracellular glutamate; anti-inflammatory signals (IL-10, TGF-β) enhance it (Luo, 2022). In spinal cord injury models (T10 contusion), electroacupuncture at Huatuojiaji (EX-B2) suppressed NF-κB while activating STAT6, shifting microglia from M1 to M2 (Li et al., 2025). In ischemic stroke models, it boosted IL-33/ST2 signaling to favor neuroprotective M2 polarization and curb inflammation (Zhang Y. et al., 2025) (Figure 4).

Crucially, the regulation of these glial cells is inextricably linked to cardiovascular function and blood pressure control. In autonomic integration centers such as the PVN and RVLM, excessive extracellular glutamate is a primary driver of sympathetic overactivity, a hallmark of hypertension (Li and Pan, 2007). Pro-inflammatory M1 microglia release cytokines (e.g., IL-1β, TNF-α) that not only directly excite presympathetic neurons but also suppress astrocytic EAAT expression, compounding glutamate accumulation (Luo, 2022). By promoting the protective M2 microglial phenotype and upregulating astrocytic EAAT1/2, acupuncture effectively clears excess synaptic glutamate and dampens neuroinflammation in these critical hubs. This coordinated glial-neuronal modulation removes the aberrant excitatory drive, thereby reducing central sympathetic outflow, lowering peripheral vascular resistance, and ultimately exerting a robust antihypertensive effect.

#### 3.2.2 Acupuncture modulates GABA concentration in the synaptic cleft

GABA functions as the main inhibitory neurotransmitter in the central nervous system, mediating a large portion of synaptic inhibition throughout the brain. Neurons produce it from glutamate through the enzyme glutamate decarboxylase (GAD), then package it into vesicles at inhibitory terminals. Release into the cleft quickly engages GABAA and GABAB receptors, promoting chloride or potassium entry to hyperpolarize neurons and curb excitation (Ganguly et al., 2001). In GABA breakdown, γ-aminobutyric acid transaminase (GABA-T) and succinic semialdehyde dehydrogenase (SSADH) collaborate. Astrocytes take up GABA, where GABA-T converts it to succinic semialdehyde, which SSADH then oxidizes to succinate for entry into the Krebs cycle and energy production.

GAD depends on vitamin B_6_ (pyridoxal phosphate) as a cofactor to decarboxylate glutamate specifically for GABA production—the sole brain pathway for this synthesis. Mammals express two isoforms: GAD65, abundant in synapses and pancreatic cells, and GAD67, widespread in GABAergic neurons. Factors like calcium levels, pH, and phosphorylation modulate GAD activity to control synthesis rates. In focal cerebral ischemia models using middle cerebral artery blockage, electroacupuncture along Yin and Yang Heel Vessel points lowered CNS release of GABA-T and SSADH, delaying breakdown, raising synaptic GABA, and strengthening inhibition (Mao et al., 2018). Post-stroke spasticity studies in rats showed acupuncture at Zusanli (ST36) elevated brain GABA levels and expression while suppressing GABA-T (Wang et al., 2020).

**Figure 5:**
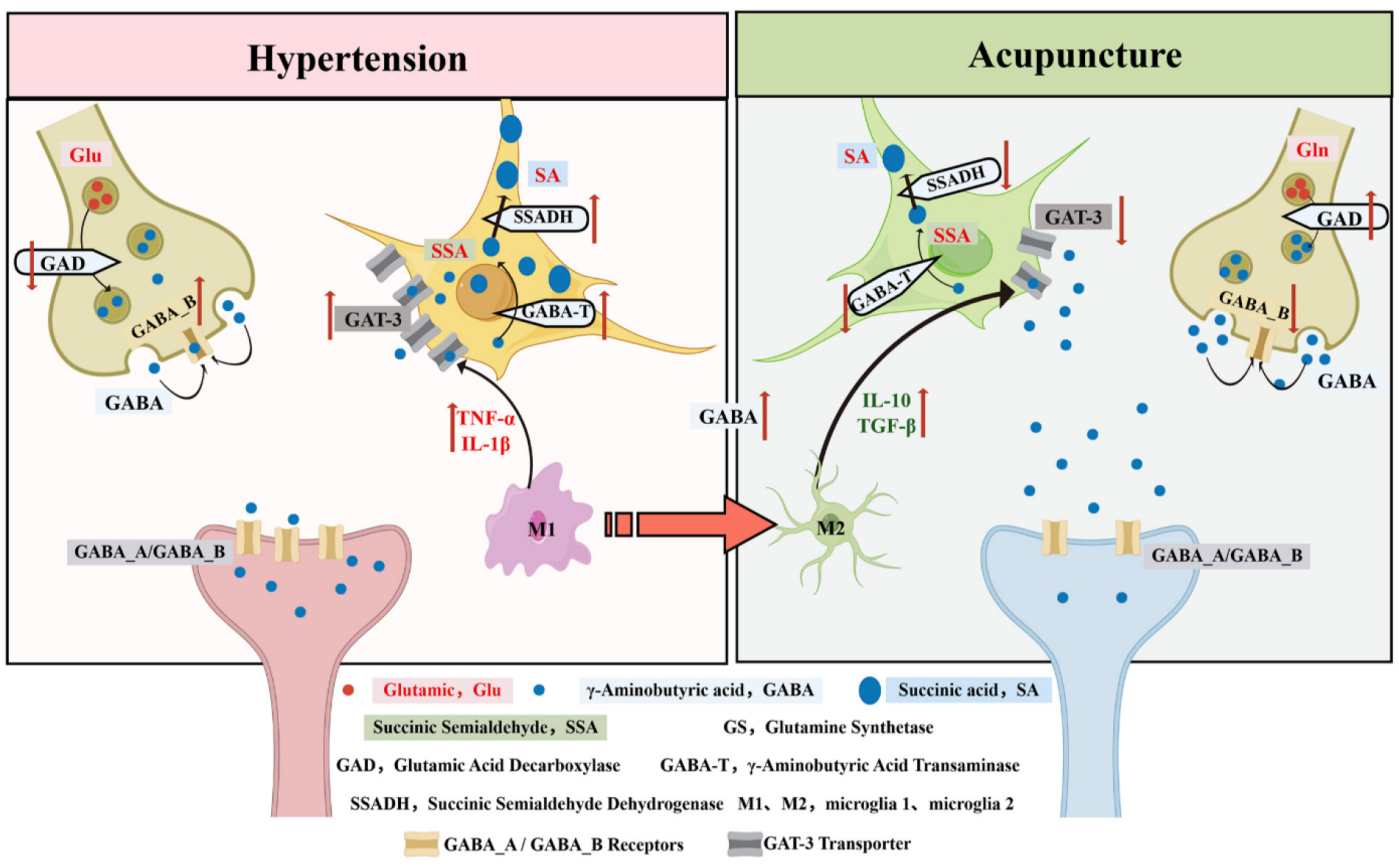
GABAergic mechanisms of acupuncture: selective activation of GABA synthesis and transport (GAD, GABA-T, GAT-3), enhancement of GABAA/GABAB receptor functions, inhibition of GABA metabolic disorder and M1 microglia-mediated inflammatory response, and restoration of inhibitory synaptic transmission in hypertensive state.

In middle cerebral artery occlusion (MCAO) models, electroacupuncture at Dazhui (GV14), Jizhong (GV6), and Houhui (anteromedial to the sixth lumbar transverse process) significantly upregulated cortical GAD67 protein and mRNA expression while downregulating GABA-T levels, thereby enhancing GABAergic inhibitory transmission (Li J. et al., 2022). Similar to the glutamatergic system, this astrocyte-mediated preservation of synaptic GABA is highly relevant to cardiovascular regulation. In autonomic control centers such as the RVLM, GABAergic inhibitory inputs act as a critical “brake” against sympathetic overactivity. By downregulating GABA-degrading enzymes (such as GABA-T and SSADH) in these medullary regions, acupuncture effectively prolongs the half-life and elevates the concentration of GABA in the synaptic cleft. This enhanced GABAergic transmission strongly hyperpolarizes presympathetic neurons, thereby safely curtailing sympathetic outflow, reducing vascular resistance, and exerting a significant antihypertensive effect (Guyenet et al., 2018; Menezes and Fontes, 2007) (Figure 5).

## 4 Acupuncture regulates the NTS-CVLM-RVLM neural circuit

Key brain structures, including the median preoptic nucleus (MnPO), paraventricular nucleus (PVN) of the hypothalamus, rostral ventrolateral medulla (RVLM), caudal ventrolateral medulla (CVLM), and nucleus of the solitary tract (NTS), interconnect through synapses to form circuits that modulate sympathetic nerve activity (SNA) and control blood pressure (Guyenet et al., 2020; Dampney et al., 2018; Gerlach et al., 2021). The NTS acts as the main brainstem hub for processing autonomic cardiovascular inputs. It combines peripheral sensory data with signals from other brainstem regions and higher centers, then relays integrated outputs to areas like the thalamus, PVN, and RVLM (Manuel et al., 2020; Dampney, 2017). From there, commands travel via preganglionic neurons to sympathetic ganglia (paravertebral and prevertebral), reaching peripheral autonomic branches and precisely targeting effector organs to coordinate responses across various systems (Li Y. W. et al., 2022).

### 4.1 Regulation of NTS neuronal electrical activity by acupuncture

The nucleus of the solitary tract (NTS) receives direct afferent signals from the vagus nerve and spinal pathways, along with descending projections from higher centers (Holt, 2022). It functions as the chief brainstem site for merging inputs from cardiovascular, respiratory, and visceral systems. From the NTS, axons project to regions such as the prefrontal cortex, central amygdala, hypothalamus, and ventrolateral medulla (Gasparini et al., 2020). These broad connections support central homeostasis and contribute to disorders affecting immune, gastrointestinal, neural, and cardiovascular functions (Tian et al., 2025). The solitary tract consists of primary visceral afferents from the facial, glossopharyngeal, and vagus nerves, ending in the NTS. Positioned ventrolateral to the dorsal motor nucleus of the vagus, the NTS features intricate local synaptic networks. Based on location, neurotransmitters, and receptors, researchers divide the NTS into several subregions: (1) Commissural subnucleus: Handles afferent inputs from chemoreceptors, baroreceptors, and gut. (2) Medial subnucleus: Processes afferent signals from cardiovascular and digestive sources, including baroreceptors. (3) Intermediate subnucleus: Receives afferent fiber from cardiopulmonary afferent fibers. (4) Interstitial subnucleus: Integrates inputs from lungs, trachea, and bronchi; often called the respiratory subnucleus. (5) Dorsolateral subnucleus: Manages baroreceptor and chemoreceptor data for cardiovascular reflexes. (6) Ventral subnucleus. (7) Ventrolateral subnucleus. The ventral and ventrolateral areas participate in carotid baroreflex and pulmonary stretch responses. The intermediate and commissural parts together form the “cardiovascular NTS” (Schaffar et al., 1988), where baroreceptor fibers primarily terminate. Rising arterial pressure activates peripheral baroreceptors in the aortic arch and carotid sinus. Signals travel via vagus and glossopharyngeal nerves to this cardiovascular NTS zone, depolarizing local neurons. This initiates two main depressor pathways: (1) glutamatergic NTS projections excite cholinergic parasympathetic preganglionic cells in the nucleus ambiguus (NA); and (2) similar projections activate GABAergic neurons in the caudal ventrolateral medulla (CVLM), which suppress adrenergic sympathoexcitatory cells in the rostral ventrolateral medulla (RVLM) (Yao et al., 2022; Dupont and Légat, 2020). The commissural subnucleus provides the initial brainstem relay and processing for arterial chemoreceptor signals (Finley and Katz, 1992; Totola et al., 2013). The carotid body, positioned at the common carotid bifurcation, detects arterial oxygen levels and converts chemical changes into neural impulses. These travel along the carotid sinus nerve (a glossopharyngeal branch) to the NTS, triggering reflexes that boost breathing depth and rate, cardiac output, and sympathetic activity (Iturriaga et al., 2017). Acupuncture at ST36 (Zusanli) reduces blood pressure in normal and hypertension models while elevating NTS neuronal firing rates (Liu et al., 2012). In severe inflammation models induced by lipopolysaccharide, stimulation at BL32 (Ciliao) markedly activates the NTS and enhances vagal efferent discharge, with brainstem autonomic nuclei serving as key integration sites for this acupoint's effects on somatic-autonomic regulation (Pan et al., 2023). Electroacupuncture at HT7 (Shenmen) and HT5 (Tongli) lowers heart rate and mean arterial pressure in ischemia-prone models; signals relay through the NTS to the hippocampus for processing, influencing specific NTS neuron excitability (Cui et al., 2018).

### 4.2 Regulation of RVLM neuronal electrical activity by acupuncture

The RVLM contains two main neuronal populations: C1 (catecholamine or adrenergic) and non-C1 cells (Guyenet et al., 2020). C1 neurons form the majority (50–70%) of those projecting to the spinal cord and represent one of the few central groups capable of epinephrine synthesis (Madden and Sved, 2003). These cells directly stimulate sympathetic preganglionic neurons in the spinal intermediolateral column using glutamate as their transmitter. Non-C1 neurons mainly process incoming cardiovascular reflex signals (from baroreceptors and chemoreceptors) and translate them into sympathetic outputs. The RVLM acts as a crucial hub in blood pressure control networks (Guyenet et al., 2018). In healthy states, GABAergic input from the caudal ventrolateral medulla (CVLM) provides essential restraint against excessive pressure rises. Hypertension often features weakened CVLM inhibition, allowing sustained RVLM activation. Presympathetic C1 neurons drive much of this pressure regulation, influenced not only by synaptic inputs from upstream sites but also potentially by local humoral factors. Within sympathetic circuits, the CVLM delivers the dominant inhibitory synapses to the RVLM. Signals from areas like the paraventricular nucleus (PVN) or nucleus of the solitary tract (NTS) engage CVLM neurons, which then curb RVLM output and reduce sympathetic flow (Moraes et al., 2013). The RVLM contributes significantly to various neurogenic hypertension forms (Dupont and Légat, 2020; Zhang et al., 2022). It integrates peripheral baroreceptor afferents for pressure adjustment (Dampney, 2017), typically routed first through the NTS before reaching the RVLM (Yao et al., 2022). Suppressing RVLM activity in rodents whether anesthetized or awake markedly drops blood pressure and sympathetic tone (Guyenet et al., 2018; Menezes and Fontes, 2007; Geraldes et al., 2014), by silencing excitatory cells with direct spinal projections. Advanced techniques like optogenetics or chemogenetics targeting C1 neurons similarly produce robust depressor effects (Wenker et al., 2017; Moraes et al., 2017). To date, however, no studies have examined direct RVLM neuronal inhibition by acupuncture as a mechanism for blood pressure reduction.

## 5 Acupuncture regulates the HPA axis to balance autonomic nervous function

The hypothalamic-pituitary-adrenal (HPA) axis serves as the primary neuroendocrine pathway for managing stress responses in the body. During stress, neurons from various sensitive brain areas project to the paraventricular nucleus (PVN) of the hypothalamus. This stimulates parvocellular neurons to release corticotropin-releasing hormone (CRH) and magnocellular neurons to produce arginine vasopressin (AVP). These peptides travel through the portal system to the anterior pituitary (Burford et al., 2017). There, CRH and AVP work together, binding to CRH receptor 1 (CRHR1) and vasopressin V1b receptor, to prompt the secretion of proopiomelanocortin (POMC). Enzymes then process POMC into adrenocorticotropic hormone (ACTH) and other peptides like β-endorphin. Circulating ACTH reaches the adrenal cortex's zona fasciculata, engaging melanocortin 2 receptors (MC2R) to boost corticosteroid (CORT, mainly cortisol) production via key enzymatic steps (Zheng J. Y. et al., 2024; Papadimitriou and Priftis, 2009). Cortisol exerts broad effects peripherally and centrally by activating glucocorticoid receptors (GR), orchestrating the stress adaptation. Rising cortisol levels normally suppress further CRH and ACTH release through negative feedback at the hypothalamus and pituitary, guarding against excessive HPA activation (Harno et al., 2018). In hypertension, the HPA axis frequently shows disrupted patterns. Prolonged stressors such as ongoing psychological strain or unfavorable conditions cause persistent PVN stimulation, altering CRH secretion rhythms and often blunting nighttime declines (Yunus et al., 2020). This drives excess pituitary ACTH output, leading to chronic high cortisol from the adrenals. A major problem arises from impaired negative feedback, allowing unchecked axis overdrive.

### 5.1 Acupuncture can reduce the expression level of CRH in the PVN

**Figure 6:**
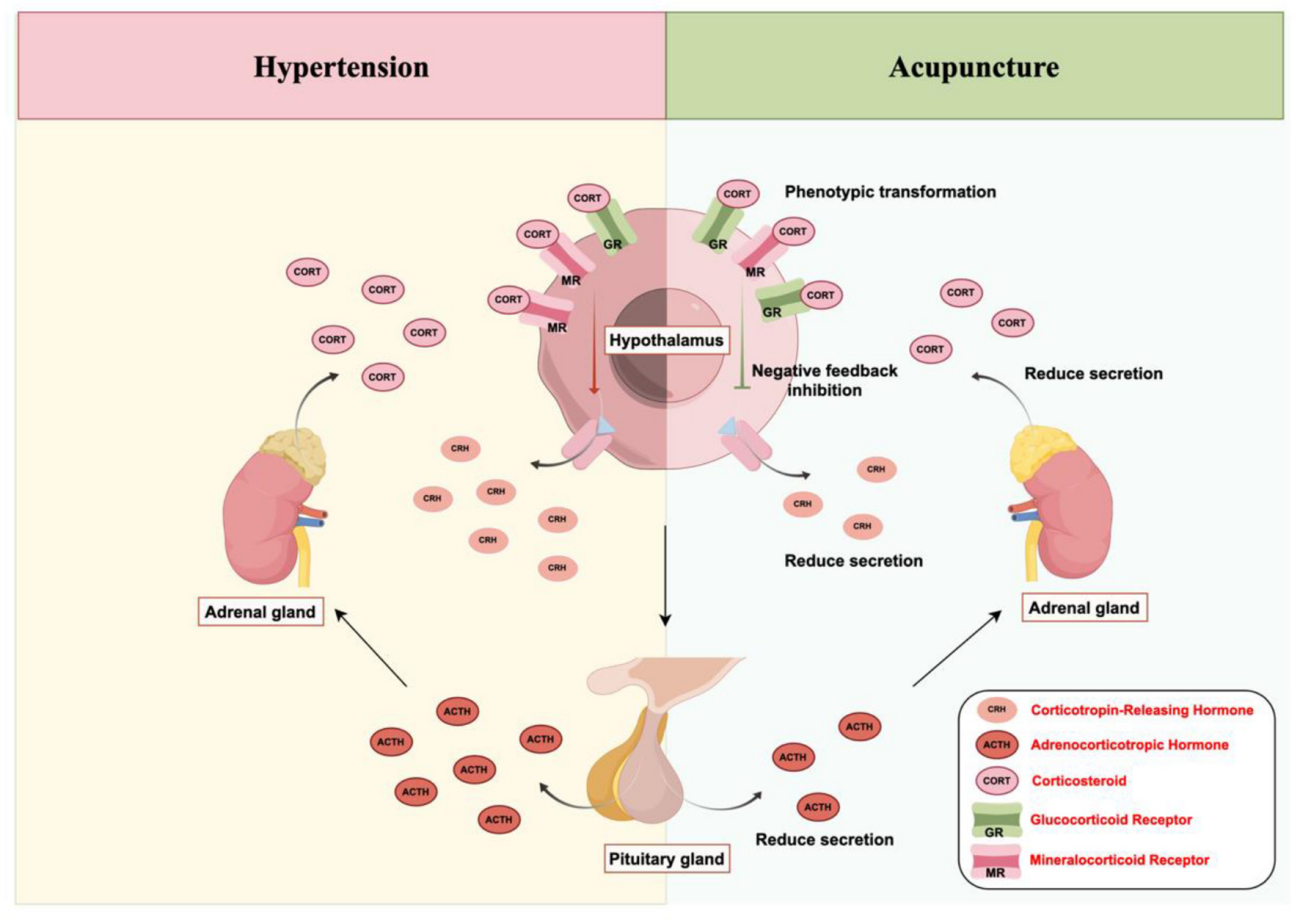
Hypothalamic-pituitary-adrenal (HPA) axis: negative feedback remodeling mechanism of acupuncture: enhancement of glucocorticoid negative feedback mediated by hypothalamic glucocorticoid receptors (GR)/mineralocorticoid receptors (MR), inhibition of the corticotropin-releasing hormone (CRH) → adrenocorticotropic hormone (ACTH) → cortisol (CORT) cascade secretion, and reduction of adrenocortical hormone output to alleviate hypertension-associated stress hyperactivity.

The paraventricular nucleus (PVN) of the hypothalamus integrates neuroendocrine and autonomic processes, exerting major control over hormone release and cardiovascular sympathetic activity (Li and Pan, 2007; Carmichael and Wainford, 2015). PVN parvocellular projections reach brainstem and spinal autonomic centers, driving sympathetic regulation of heart rate (Pyner, 2014). Corticotrophin-releasing hormone (CRH) neurons drive neuroendocrine stress reactions, with dense expression in the PVN alongside sites like the amygdala, arcuate nucleus, supraoptic nucleus, locus coeruleus, and cortex (Wang et al., 2010). CRH blockers suppress about 70% of acute stress-triggered ACTH surges, underscoring its dominance in HPA activation. PVN CRH excess heightens both sympathetic tone and HPA output (Rivier and Plotsky, 1986; Goncharuk et al., 2002). Such disruptions link to cardiovascular pathologies like hypertension, ischemia, and irregular rhythms through altered sympathetic input (Goncharuk et al., 2007). CREB transcription factor governs CRH gene activity. PVN Nesfatin-1 elevation triggers the ERK/CREB route, boosting CRH production (Bai et al., 2025). Electroacupuncture at Zusanli (ST36) and Sanyinjiao (SP6) counters this by curbing ERK/CREB phosphorylation and CRH levels (Zheng et al., 2024), confirming suppression of CRH via this cascade. In unpredictable chronic mild stress rats, specific acupoint needling, unlike sham sites, eased behaviors, cut blood CORT, raised PVN GR, and curbed CRH overproduction (Wang et al., 2015). Post-hepatectomy models displayed PVN CRH mRNA/protein spikes without treatment; electroacupuncture at Zusanli (ST36) and Shangjuxu (ST37) sharply reduced them, along with HPA markers such as ACTH and CORT (Wang et al., 2023) (Figure 6).

### 5.2 Acupuncture can regulate the synthesis of POMC in the anterior pituitary and the secretion of ACTH

Pro-opiomelanocortin (POMC) is a key precursor protein produced mainly in anterior pituitary corticotrophs and hypothalamic arcuate nucleus neurons, as well as in other sites. Specific convertase enzymes process it into active peptides, including adrenocorticotropic hormone (ACTH), β-endorphin, and α-, β-, and γ-melanocyte-stimulating hormones. These fragments influence diverse functions: energy balance, HPA axis control, reproduction, thermoregulation, pain processing, glandular secretion, immunity, and pigmentation (Jiang et al., 2025; Jenks, 2009; Böhm and Grässel, 2012). ACTH drives glucocorticoid production in stress responses, with its output largely governed by POMC gene activity and protein synthesis in pituitary cells. Corticotropin-releasing hormone (CRH) engages pituitary receptors, stimulating Gs-coupled adenylyl cyclase to raise cyclic AMP levels and activate protein kinase A (PKA) (Drouin, 2016). PKA's catalytic parts phosphorylate CREB and coactivators like CRTC2/3 in the nucleus. The complex binds cAMP response elements on the POMC promoter, boosting transcription (Drouin, 2016; Bousquet et al., 2000). Newly made POMC travels through the endoplasmic reticulum and Golgi, entering secretory granules where PC1/3 cleaves it to form ACTH for storage in corticotroph vesicles (Wang et al., 2017; Lightman et al., 2020). Subsequent CRH signaling depolarizes membranes and elevates calcium, prompting vesicle fusion and ACTH release. Circulating ACTH quickly engages MC2R in areas like the paraventricular nucleus (PVN) and nucleus of the solitary tract (NTS), activating sympathetic premotor cells non-genomically to increase sympathetic drive (Fridmanis et al., 2017). In hypertension models, prolonged ACTH elevation fuels a feedback loop with PVN oxidative stress and inflammation: high ACTH promotes local damage, which heightens sympathetic overactivity (Henry et al., 2012). Acupuncture-triggered endogenous opioids, especially β-endorphin (Bae et al., 2025), likely temper vesicle release and POMC/ACTH excitation via local feedback (Wu et al., 2023). Electroacupuncture at ST36 (Zusanli) and SP6 (Sanyinjiao) cut plasma CRH, ACTH, and corticosterone by 30–40% in chronic stress rats (Zhou and Longhurst, 2012). Combining points like DU20 (Baihui), HT7 (Shenmen), and LR3 (Taichong) in depression models dropped ACTH around 25% and enhanced hippocampal glucocorticoid receptor levels. These effects point to acupuncture's broad tuning of the POMC/ACTH system by dampening HPA overdrive and strengthening negative feedback (Gong et al., 2021) (Figure 6).

### 5.3 Acupuncture regulates MR/GR expression and CORT secretion

Cortisol exerts a dual influence on the autonomic nervous system at central levels. Prolonged stress sustains HPA axis activity, raising circulating cortisol that cooperates with central sympathetic pathways to heighten excitability and elevate blood pressure. Conversely, cortisol engages mineralocorticoid receptors (MR) and glucocorticoid receptors (GR) in the brain to enforce negative feedback, restraining both HPA overdrive and sympathetic outflow, thus capping pressure surges under some circumstances (Koning et al., 2019; de Kloet et al., 2005, 2018). Essential hypertension often arises from chronic disruption of this balance, where protective mechanisms fail. The brain hosts two cortisol receptor types with distinct roles: GR, abundant in the hypothalamus, hippocampus, cortex, and brainstem, primarily transmit stress-related glucocorticoid signals; MR, concentrated in the hippocampus, amygdala, and select hypothalamic areas, dominate under resting cortisol conditions to support mood stability, stress readiness, and baseline cardiovascular control (Koning et al., 2019). Enzymes 11β-hydroxysteroid dehydrogenase type 1 (11β-HSD1) and type 2 (11β-HSD2) fine-tune local active cortisol through interconversion with inert cortisone, creating pre-receptor control. 11β-HSD1 mainly regenerates cortisol and expresses strongly in the hippocampus, amygdala, cortex, cerebellum, and hypothalamic PVN, amplifying regional signaling (Zhang et al., 2006; Seckl, 2024; Jiang and Chen, 2005). 11β-HSD2 inactivates cortisol, appearing selectively in the NTS and ventromedial hypothalamus to shield neurons from excess exposure by blocking MR activation (Jiang and Chen, 2005; Chapman et al., 2013; Luft, 2016). Blocking 11β-HSD2 in the PVN raises mean arterial pressure and sympathetic tone, underscoring its restraining role (Zhang et al., 2006). Shifts in GR/MR binding and 11β-HSD1/2 activities across regions like the PVN, NTS, and hippocampus set the intensity and triggers for stress-driven cardiovascular responses. The PVN integrates HPA initiation (via CRH cells) with sympathetic preganglionic control. Targeted GR reduction in PVN using CRISPR/Cas9 in rats increased CRH neuron firing and portal CRH delivery, paradoxically elevating peripheral cortisol through lost feedback (Zhang et al., 2024a; Zhang H. et al., 2025). This mismatch between central GR deficit and systemic hypercortisolemia underlies concurrent sympathetic and HPA hyperactivity in essential hypertension (Zhang et al., 2024a). NTS 11β-HSD2 levels shape baroreflex gain (Seckl, 2024). Overactive NTS MR dampens the reflex, raising pressure variability (Takahashi et al., 2011). Diminished NTS 11β-HSD2 in salt-sensitive models permits local cortisol buildup, aberrant MR signaling, baroreflex resetting, and sympathetic threshold shifts that favor hypertension progression (Seckl, 2024). The hippocampus, rich in both receptors, links stress memory to cardiovascular modulation. Chronic stress desensitizes hippocampal GR, sparking NLRP3 inflammasome activity, IL-1β release, and distant PVN excitation a “hippocampal inflammation to hypothalamic drive” loop. Regional 11β-HSD1 overexpression in CA3 heightens stress-induced pressure responses, while knockout blunts them (Min et al., 2023). Thus, the hippocampal 11β-HSD1–GR pathway influences vulnerability to stress-linked hypertension, aligning with emotional triggers and sympathetic excess in many patients. Acupuncture at Shenshu (BL23) and Qimen (LR14) curbs upstream CRH drivers and boosts GR expression or responsiveness (Wang et al., 2014), strengthening cortisol's inhibitory feedback on the HPA axis for layered control. Hypertensive individuals often show defective cortisol-GR interaction; acupuncture appears to restore HPA responsiveness by enhancing GR performance (Mulatero et al., 1997). Electroacupuncture at ST36 and SP6 markedly lowered hypothalamic urocortin 1 and circulating CORT in stress models (Zhang et al., 2010). In anesthetized rats, LI4 stimulation blocked dental pain-induced ACTH and CORT rises (Han et al., 1999). In PTSD-like rats, electroacupuncture at DU20 and ST36 reduced hippocampal MR while raising GR, suggesting broad repair of stressed neurons and rebalanced MR/GR ratios to normalize HPA function (Hou et al., 2013) (Figure 6).

## 6 Discussion

This review comprehensively explores how acupuncture restores blood pressure balance by modulating autonomic nervous system (ANS) function through an intricate, multi-target, multi-pathway, and multi-level network. Through synthesis of current evidence, it maps a pathway from peripheral detection to central processing, revealing acupuncture's antihypertensive action as a coordinated systemic adjustment. The process begins with targeted physical input at acupoints, activating select peripheral nerves that relay signals centrally, amplifying regulatory cascades to reestablish autonomic equilibrium. Acupuncture influence on the ANS unfolds in three phases: sensation, transmission, and effect. Sensation involves the local detection of needle stimulation at acupoints, where acupuncture (including electroacupuncture) activates sensory nerve fibers through mechanical, chemical, and electrical stimulation of afferent pathways (Zhou et al., 2005), ultimately transforming these inputs into neural signals. Acupoint microenvironments share features but differ by site and stimulation parameters. Effect encompasses responses in target tissues, varying by condition.

Transmission relies on neural routes carrying signals from acupoints to organs (Longhurst, 2010). Acupuncture excites peripheral afferents, routing information via spinal and cranial paths to the brain for integration, then influencing effectors through efferent nerves or neurohormonal routes (Torres-Rosas et al., 2014; Liu et al., 2020; Ulloa, 2021, 2023). Selective recruitment of peripheral fibers acts as the trigger for therapeutic outcomes. Acupuncture preferentially engages Aβ, Aδ, and C fibers based on intensity, method, and point choice, rather than broad activation (Jing and Zhu, 2024). Fast-conducting Aβ mechanoreceptors likely handle initial touch and signal relay. Multimodal Aδ and C fibers, linked to pain and temperature sensing, transmit deeper regulatory inputs. Autonomic adjustments emerge prominently when stimulation surpasses Aδ and/or C fiber thresholds, with stronger intensity yielding greater responses. This fiber-specific engagement converts basic mechanical cues into nuanced bioelectrical patterns, enabling refined central control. At central synapses, acupuncture optimizes transmission by adjusting neurotransmitter dynamics, receptor responsiveness, glial support, and local conditions (Voutsinos-Porche et al., 2003). It elevates synaptic plasticity markers in critical areas like the nucleus of the solitary tract, dorsal raphe, and hypothalamus, promoting long-term potentiation and better circuit integration. This targeted tuning favors endogenous controls, such as descending inhibition and autonomic hubs, supporting coordinated management of pain, immunity, and metabolism. Central processing distributes across interconnected nodes, including the NTS, CVLM, RVLM, and PVN. The NTS, primary gateway for cardiovascular afferents, merges baroreceptor and chemoreceptor data. Acupuncture boosts NTS neuronal firing, strengthening upstream influence. Downstream antihypertensive actions proceed via two routes: activating CVLM GABAergic cells to suppress RVLM sympathetic drive, and imposing direct or indirect restraint on RVLM presympathetic neurons. As the main sympathetic output station, RVLM suppression lowers peripheral sympathetic tone and blood pressure. Precise orchestration of the NTS-CVLM-RVLM loop forms the neurophysiological cornerstone of acupuncture's depressor effects. Chronic stress hyperactivates the HPA axis the central stress mediator fueling persistent sympathetic elevation and hypertension. Overactive PVN CRH neurons sustain high ACTH and cortisol release, compounded by impaired glucocorticoid receptor (GR) feedback. Acupuncture counters this dysregulation across stages. Upstream, it blocks ERK/CREB signaling to curb PVN CRH gene and protein output, damping stress initiation. Midstream, it prompts endogenous opioids like β-endorphin, potentially limiting pituitary POMC processing and ACTH secretion via local feedback. Downstream, it lowers circulating cortisol while elevating GR in regions like the hippocampus, reinforcing negative feedback and rebuilding HPA self-control.

From a broader perspective, the intricate autonomic mechanisms elucidated in animal models provide a robust foundation for translational medicine. Identifying specific afferent fiber thresholds (e.g., Aδ and C fibers) and their target central nuclei directly informs the optimization of clinical parameters. Translating the stimulus-intensity dependency observed *in vivo* into standardized electroacupuncture protocols can significantly enhance clinical trial designs for hypertension. Furthermore, these neuroanatomical insights are inspiring the development of novel targeted neuromodulation devices, which utilize precise somatic stimulation to recruit autonomic reflexes akin to traditional acupuncture. Clinically, this translational potential is increasingly supported by global evidence beyond Chinese populations. Recent randomized controlled trials conducted in diverse ethnic cohorts have demonstrated that acupuncture consistently modulates autonomic tone and lowers blood pressure. For instance, a rigorously designed trial in a German cohort showed that a 6-week course of traditional acupuncture significantly lowered 24-h ambulatory systolic and diastolic blood pressures in patients with uncomplicated hypertension (Flachskampf et al., 2007). Similarly, a clinical study in Thailand reported that integrating acupuncture with standard antihypertensive medication (amlodipine) yielded superior blood pressure control compared to medication alone (Vilaval et al., 2019). This cross-cultural validation underscores that acupuncture's autonomic remodeling is a universal physiological response rather than a population-specific phenomenon, highlighting its immense translational value in global cardiovascular management.

Acupuncture has gained substantial ground in evidence-based practice and integration into modern care. The 2019 World Health Organization report on traditional and complementary medicine noted that, among responding member states from 2012–2018, most incorporated acupuncture (Huang et al., 2021). The organization recognizes hypertension as suitable for acupuncture, mainly as a low-risk adjunct that, alongside standard therapy, aids pressure control, symptom relief, and better daily functioning.

## 7 Conclusion

In summary, this paper offers a comprehensive analysis of how acupuncture addresses the imbalance of ANS homeostasis in hypertension, highlighting its holistic role in managing high blood pressure. By explaining how acupuncture influences various biological processes, it demonstrates its potential effects on peripheral nerve initiation, synaptic neurotransmitter receptors, and central nervous system regulation. We emphasize that acupuncture-induced blood pressure reduction is not due to a single cellular or molecular component, but rather a coordinated process initiated by peripheral nerves, integrated and amplified within central neural networks. This multifaceted, multi-level integration of autonomic signaling provides a robust mechanistic framework for the clinical application of acupuncture in managing hypertension.
